# Encoding of speech sounds at auditory brainstem level in good and poor hearing aid performers^☆^

**DOI:** 10.1016/j.bjorl.2016.06.004

**Published:** 2016-07-14

**Authors:** Hemanth Narayan Shetty, Manjula Puttabasappa

**Affiliations:** All India Institute of Speech and Hearing, Department of Audiology, Mysuru, Karnataka, India

**Keywords:** Frequency following response, Acceptable noise level, Hearing aid performer, Frequência seguida de resposta, Nível de ruído aceitável, Usuário de aparelho auditivo

## Abstract

**Introduction:**

Hearing aids are prescribed to alleviate loss of audibility. It has been reported that about 31% of hearing aid users reject their own hearing aid because of annoyance towards background noise. The reason for dissatisfaction can be located anywhere from the hearing aid microphone till the integrity of neurons along the auditory pathway.

**Objectives:**

To measure spectra from the output of hearing aid at the ear canal level and frequency following response recorded at the auditory brainstem from individuals with hearing impairment.

**Methods:**

A total of sixty participants having moderate sensorineural hearing impairment with age range from 15 to 65 years were involved. Each participant was classified as either Good or Poor Hearing aid Performers based on acceptable noise level measure. Stimuli /da/ and /si/ were presented through loudspeaker at 65 dB SPL. At the ear canal, the spectra were measured in the unaided and aided conditions. At auditory brainstem, frequency following response were recorded to the same stimuli from the participants.

**Results:**

Spectrum measured in each condition at ear canal was same in good hearing aid performers and poor hearing aid performers. At brainstem level, better *F*_0_ encoding; *F*_0_ and *F*_1_ energies were significantly higher in good hearing aid performers than in poor hearing aid performers. Though the hearing aid spectra were almost same between good hearing aid performers and poor hearing aid performers, subtle physiological variations exist at the auditory brainstem.

**Conclusion:**

The result of the present study suggests that neural encoding of speech sound at the brainstem level might be mediated distinctly in good hearing aid performers from that of poor hearing aid performers. Thus, it can be inferred that subtle physiological changes are evident at the auditory brainstem in a person who is willing to accept noise from those who are not willing to accept noise.

## Resumo

**Introdução:**

Os aparelhos auditivos são prescritos para aliviar a perda de audibilidade. Tem sido relatado que cerca de 31% dos usuários rejeitam seu próprio aparelho auditivo devido ao desconforto com o ruído de fundo. A razão para a insatisfação pode estar situada em qualquer local desde o microfone do aparelho auditivo até a integridade de neurônios ao longo da via auditiva.

**Objetivos:**

Medir espectros desde a saída do aparelho auditivo no nível do canal auditivo externo e frequência de resposta (FFR) registrada no tronco encefálico de indivíduos com deficiência auditiva.

**Método:**

Um total de sessenta participantes com deficiência auditiva neurossensorial moderada, com faixa etária de 15 a 65 anos foram envolvidos. Cada participante foi classificado como usuário bom ou mau de prótese auditiva (GHP ou PHP) com base na medida de nível de ruído aceitável (ANL). Estímulos /da / e /si/ foram apresentados em alto-falante a 65 dB SPL. No meato acústico externo, os espectros foram medidos nas condições sem aparelho e com aparelho. No tronco encefálico auditivo, FFR foram registradas para os mesmos estímulos dos participantes.

**Resultados:**

Os espectros medidos em cada condição no canal auditivo foram os mesmos em GHP e PHP. No nível do tronco cerebral, melhor codificação F0; energias de F0 e F1 foram significativamente maiores em GHP do que em PHP. Embora os espectros do aparelho auditivo fossem quase os mesmos entre GHP e PHP, existem variações fisiológicas sutis no tronco encefálico auditivo.

**Conclusão:**

O resultado do presente estudo sugere que a codificação neural do som da fala no nível do tronco encefálico pode ser mediada distintamente em GHP em comparação com PHP. Assim, pode-se inferir que mudanças fisiológicas sutis são evidentes no tronco encefálico em uma pessoa que está disposta a aceitar o ruído em comparação com aqueles que não estão dispostos a aceitar o ruído.

## Introduction

Hearing aid is one of the common rehabilitative measures for individuals with permanent hearing impairment. In some cases of hearing losses, hearing aids can be used transitory. However, hearing aid users often complain of background noise resulting in rejection of hearing AID.^1^ Kochkin^2^ has reported that about 31% of the hearing aid users reject their hearing aid because of background noise. Various outcome measures are available that consider background noise as one of the factors to have an effect on satisfaction with the hearing aid. Unfortunately, these outcome measures are to be administered after a period of experience with hearing aid. Besides measures such as speech in noise test, quick speech in noise test, competing noise test, and hearing in noise test are being used to predict the hearing aid benefit.^3^ Though these tests are sensitive to measure speech performance in noise and are administered at the time of fitting hearing aid; they fail to predict real-world benefit and/or satisfaction from hearing Aids.^4^ This issue is addressed partly by acceptable noise level (ANL) measure introduced by Nabelek et al.,^5^ in which the client rates annoyance due to background noise in the presence of speech.

Nabelek et al.^6^ demonstrated that value of ANLs predict good and poor hearing aid performers with 85% accuracy. The ANL is not affected by the type of background noise,^5^ preference of background sounds,^7^ primary language of the listener,^8^ speech presentation levels,^7^ age, hearing sensitivity and language^9^ content of speech signal and speaker gender.^10^ Harkrider,^11^ studied the physiological correlate of ANL involved at higher auditory centres utilizing electrophysiological measurement. In individuals with low ANLs (i.e., greater background noise acceptance), amplitudes of wave V of auditory brainstem response (ABR), all components of middle latency response (MLR), and late latency response (LLR) were noted to be significantly prolonged when compared to individuals who obtained high ANLs (lower background noise acceptance). This is due to stronger efferent mechanism, such that sensory inputs are suppressed and/or central afferent mechanism is less active.^12^ Thus, ANL proved to be physiologically sensitive measure. However, it is interesting to know the way in which amplified speech is represented physiologically in good and poor hearing aid performers.

Despite advancement in hearing aid technology, some individuals accept hearing aid and others may reject in spite of fact that they have a similar hearing loss in terms of degree, type, and configuration. The variability in satisfaction from rehabilitative device might probably be due to the processing parameters of hearing aid, and/or at interaction between output of hearing aid and its acoustic parameters relayed through different parts of auditory pathway.^13^ In the present study, output of hearing aid is investigated at ear canal and at auditory brainstem.

Over the decades, researchers have used probe tube microphone (PTM) system to measure the effect of hearing aid processing on acoustics of speech. The PTM measurement reflects the acoustic effect of the factors such as pinna, ear canal, head and torso.^14^ Primarily, the PTM is used to optimize/verify the hearing aid gain to match with the target gain at different frequencies as prescribed by the fitting formula.^15^ It is well established that output of hearing aid at the ear canal will alter amplitudes of formants leading to misperception. An experiment was conducted by Stelmachowicz et al.^16^ who recorded output of the hearing aid at the ear canal using linear and non-linear hearing aids on three listeners with mild-moderate sensorineural hearing loss. They carried out spectral analysis on these recorded stimuli. The results revealed a precipitous roll-off in the high frequency response, thus limiting the information of a consonant cues. On similar line, Souza and Tremblay^17^ conducted a study to correlate consonant errors to acoustic analysis of amplified speech in subjects with mild to moderate sensorineural hearing loss. They observed that /da/ stimulus was consistently misperceived as /ga/. This was attributed to the amplitude of aided burst spectrum of /da/ which was found to be similar to the unprocessed burst spectrum amplitude of /ga/. Kewley-Port^18^ reported that identification of stop consonants in the initial position requires the spectrum of burst as the primary cue for speech recognition. Thus, after amplification the stop consonants are more likely to have place error.^17^ However, amplified consonant-vowel combination of fricative or affricative tends to show manner errors, as consistent misperception of /ʓi/ for /dʓi/ speech sounds^19^ was noted. When the acoustic output of hearing aid was analyzed, it was revealed that amplitude spectrum of fricative /ʓi/ was similar to the unprocessed affricative spectrum amplitude of /dʓi/. Hence, performing spectral analysis of the output of hearing aid recorded at the ear canal throws light on the processing parameters of hearing aid. There are instances in which acoustic cues are distorted but a listener still recognizes correctly. This could be due to redundancy or from the contextual cues of speech. In some other instances acoustic cues are preserved but a listener fails to recognize speech sound. This may possibly be because of insufficient sensitivity in cochlea and/or concomitant changes at different levels of auditory pathway. Hence, an evoked potential recorded to speech stimuli should be used to validate perception registered at different levels of auditory pathway. In the present study, evoked response at the level of auditory brainstem of good and poor hearing aid performers is investigated.

The frequency following responses (FFR) has been extensively studied to understand the physiological processing of speech at the auditory brainstem level. The FFR is a phase-locked response to periodic aspects of stimuli, including speech, up to 1000 Hz^20^, ^21^ from the neural population of inferior colliculus of rostral brainstem.^22^ The FFR has been reliably recorded to consonant-vowel sounds /da/.^23^, ^24^, ^25^, ^26^ Further, FFR to /da/ stimulus has been investigated in monaural^27^ and binaural^28^ conditions; in the presence of background noise;^27^ and stimulation of either right or left ear.^29^ The FFR was successfully recorded using loudspeaker as a transducer to deliver the stimuli /da/ and /si/.^30^ From this, it is clear that FFR is a stimulus contingent response that is most robust for mid- and low-frequencies. Though the frequency response of hearing aid is up to 6500 Hz, the FFR is a sensitive tool to any change in processing in the auditory brainstem, such that it answers the question on how amplified speech sounds are encoded in mid- and low-frequencies from good and poor hearing aid performers.

From the existing literature, it can be inferred that spectral analyses of hearing aid output obtained using the PTM give information on hearing aid processing at the ear canal level. Further, stimulus from the ear canal is relayed to the auditory brainstem level and is measured using FFR. The FFR will help in inferring the neural encoding of the ongoing speech. These measures give insight into the way in which the speech is neurally encoded at the brainstem level, in individuals with sensorineural hearing impairment who are classified as good and poor hearing aid performers having comparable type, degree and configuration of hearing loss. Therefore, the present study intends to investigate hearing aid output at the ear canal to determine extent of alteration caused by the hearing aid on the spectral parameters. In addition, way in which amplified speech is represented at the brainstem level in good and poor hearing aid performers also is being investigated. The objectives formulated for the study were to compare: (1) spectral changes between GHP and PHP in unaided and aided conditions at the ear canal using the PTM; and (2) neural encoding of speech sounds at auditory brainstem level in GHP and PHP.

## Methods

### Participants

**Figure 1:**
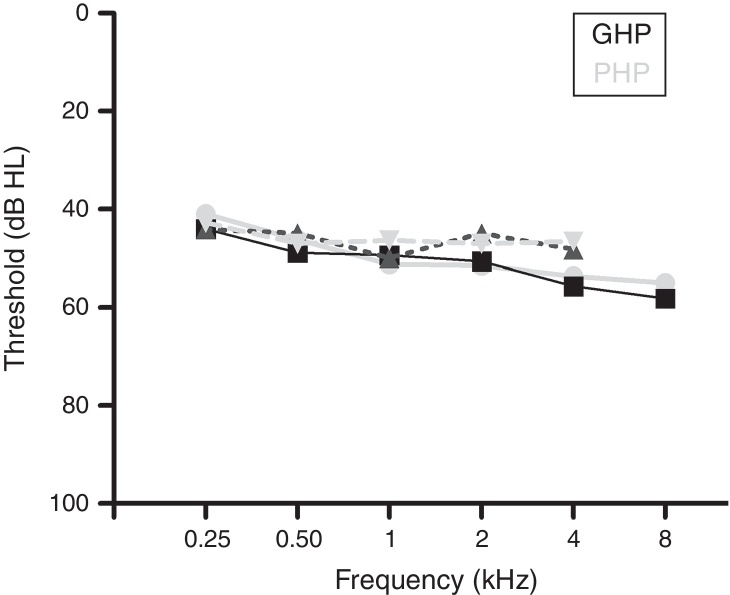
Audiograms of good and poor hearing aid performers.

A total of 60 participants who had bilateral moderate sensorineural hearing loss with a flat configuration were involved in the study. Flat configuration was operationally defined as the difference between least and highest air-conduction thresholds being less than 20 dB in the range from 0.25 to 8 kHz.^31^ The age range of the participants was from 15 to 65 years. They had speech identification scores (SIS) that was greater than or equal to 75% at 40 dB SL (re: speech reception threshold, SRT). The test ear had normal middle ear status as indicated by ‘A’ type tympanogram with middle ear peak pressure ranging from +50 daPa to −100 daPa, and admittance ranging from 0.5 mL to 1.75 mL. The auditory brainstem response (ABR) was recorded at two repetition rates of 11.1 s and 90.1 s at 90 dB nHL to ensure that there was no retro cochlear pathology. The latency difference of V peak of ABR was found to be less than 0.8 ms between the two repetition rates. All participants were naïve hearing aid users and there was no self-reported history of other otological and neurological problems. The participants were further classified into Good or Poor Hearing aid Performers (GHP or PHP) using the acceptable noise level (ANL).^6^ Those participants who obtained an ANL score of ≤7 were considered as good hearing aid performers and a score of ≥13 were considered as poor hearing aid performers.^6^ The demographic data of each participant in clinical group are tabulated in Table 1. The hearing thresholds at each audiometric frequency of the test ear of the good and poor hearing aid performers are depicted in Fig. 1. The study was approved by the All India Institute of Speech and Hearing Ethics Committee for Research in Humans. Informed consent was obtained from each participant.

**Table 1:** Demographic data of study participants.

SN	Age (yrs)	Gender	Ear	PTA dB HL	SIS (Max. 25)	V peak latency difference at two repetition rates (ms)	Grouping based on ANL	ANL scores (in dB)
1	15.00	F	R	58.30	20.00	0.38	GHP	3.00
2	18.00	M	L	45.00	21.00	0.44	GHP	6.00
3	18.00	F	R	56.60	20.00	0.35	GHP	6.00
4	19.00	M	R	48.30	21.00	0.46	GHP	6.00
5	19.00	M	L	53.30	25.00	0.27	GHP	3.00
6	25.00	F	R	55.00	21.00	0.46	PHP	14.00
7	27.00	M	L	50.00	19.00	0.65	PHP	15.00
8	27.00	F	R	51.60	19.00	0.55	PHP	15.00
9	27.00	M	R	51.60	21.00	0.25	PHP	16.00
10	32.00	M	R	53.30	20.00	0.41	GHP	6.00
11	32.00	M	R	55.30	22.00	0.33	PHP	17.00
12	35.00	M	R	51.60	23.00	0.56	GHP	6.00
13	26.00	F	R	55.00	22.00	0.30	GHP	6.00
14	37.00	M	L	51.60	21.00	0.34	PHP	14.00
15	40.00	F	L	50.00	20.00	0.30	GHP	6.00
16	41.00	M	L	50.00	20.00	0.27	PHP	17.00
17	42.00	M	L	50.00	25.00	0.37	PHP	14.00
18	43.00	M	L	55.00	20.00	0.25	GHP	6.00
19	47.00	F	R	55.60	23.00	0.38	GHP	4.00
20	47.00	M	R	46.60	19.00	0.37	PHP	14.00
21	49.00	M	R	53.30	20.00	0.38	GHP	6.00
22	50.00	F	R	53.30	23.00	0.30	PHP	14.00
23	50.00	M	L	45.00	25.00	0.55	GHP	5.00
24	53.00	M	L	45.00	21.00	0.22	PHP	13.00
25	54.00	F	R	51.60	24.00	0.39	PHP	14.00
26	54.00	M	R	48.30	24.00	0.28	GHP	5.00
27	54.00	M	L	41.00	23.00	0.30	GHP	2.00
28	54.00	M	L	41.60	23.00	0.57	GHP	4.00
29	55.00	F	L	55.00	21.00	0.24	PHP	18.00
30	55.00	F	R	53.30	23.00	0.50	GHP	3.00
31	55.00	F	L	50.00	23.00	0.58	GHP	2.00
32	51.00	M	L	45.00	24.00	0.50	GHP	6.00
33	56.00	F	L	46.60	23.00	0.24	GHP	4.00
34	58.00	M	R	45.00	24.00	0.34	GHP	6.00
35	58.00	M	L	48.30	24.00	0.32	GHP	6.00
36	58.00	M	R	45.00	24.00	0.24	GHP	4.00
37	58.00	M	L	45.00	24.00	0.34	GHP	2.00
38	60.00	F	R	41.60	21.00	0.32	GHP	6.00
39	60.00	F	L	53.30	21.00	0.22	GHP	2.00
40	60.00	F	R	45.00	24.00	0.54	PHP	15.00
41	60.00	M	R	55.00	20.00	0.43	PHP	14.00
42	60.00	M	L	43.30	25.00	0.55	GHP	3.00
43	60.00	M	R	46.30	24.00	0.37	PHP	15.00
44	60.00	M	L	46.30	24.00	0.23	PHP	14.00
45	60.00	M	R	58.30	20.00	0.34	PHP	16.00
46	61.00	F	L	55.00	23.00	0.44	GHP	5.00
47	61.00	F	R	58.30	24.00	0.36	GHP	6.00
48	61.00	M	R	41.60	24.00	0.70	PHP	16.00
49	61.00	M	R	55.00	21.00	0.42	PHP	14.00
50	62.00	M	R	55.00	19.00	0.26	GHP	2.00
51	62.00	M	L	51.60	25.00	0.28	PHP	13.00
52	62.00	F	R	50.00	24.00	0.34	PHP	14.00
53	64.00	M	L	45.00	20.00	0.23	PHP	16.00
54	64.00	F	L	55.30	22.00	0.28	GHP	5.00
55	65.00	M	L	51.60	21.50	0.28	PHP	14.00
56	65.00	M	R	55.00	24.00	0.32	GHP	5.00
57	65.00	M	R	46.60	21.50	0.30	GHP	5.00
58	65.00	M	R	43.30	20.00	0.35	GHP	6.00
59	65.00	M	R	50.00	18.00	0.68	GHP	6.00
60	65.00	F	R	56.60	24.00	0.45	PHP	14.00

HL, hearing loss; PTA, pure tone audiometry; SIS, speech identification scores; ANL, acceptable noise level; F, female; M, male; R, right ear; L, left ear; GHP, good hearing aid performers; PHP, poor hearing aid performers.

### Stimuli

**Figure 2:**
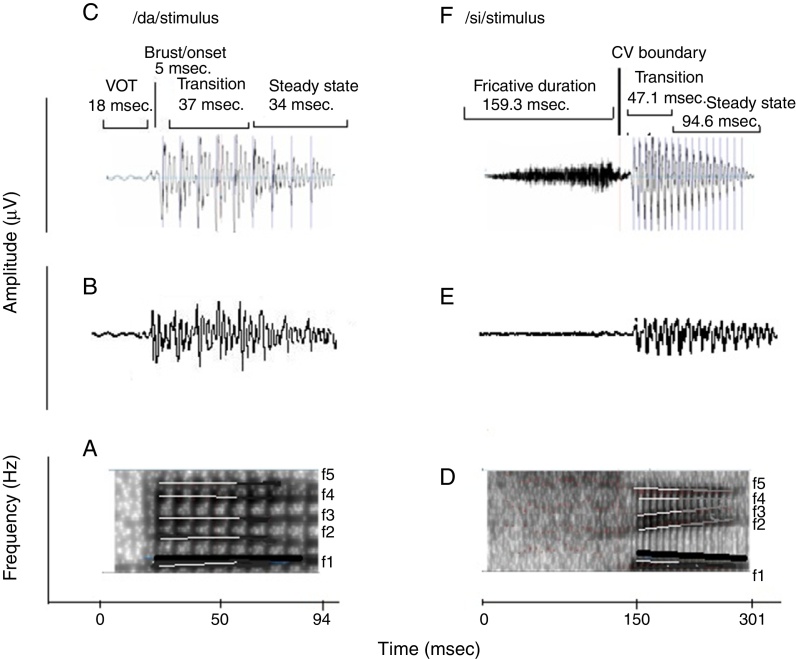
(A) and (D) are the spectrograms of /da/ and /si/ stimuli. The dark black solid line in both stimuli indicates the *F*_0_, which has a falling pattern. The formant frequencies are represented by white lines. The *F*_1_ and *F*_2_ of /da/ is flat in pattern. The *F*_1_ of /si/ stimulus is falling in pattern and *F*_2_ is raising in pattern. (C) and (F) are the waveforms of /da/ and /si/ stimuli. For sound /da/, the voice onset time was 18 ms, the burst duration was 5 ms, the transition duration was 37 ms and vowel duration of 34 ms. For the sound /si/, the fricative duration was 159.3 ms, transition duration was 47.1 ms and vowel duration was 94.6 ms.

Naturally produced consonant vowel (CV) tokens were utilized as target test stimuli. An adult male with normal voice was used to record the CV tokens. The duration of /da/ and /si/ stimuli was 94 ms and 301 ms, respectively. For /da/, the voice onset time was 18 ms, burst duration was 5 ms, transition duration was 37 ms, and vowel duration was 34 ms. For /si/, the fricative duration was 159.3 ms, transition duration was 47.1 ms and the vowel duration was 94.6 ms. Both the stimuli were converted from ‘.wav’ to ‘.avg’ format using wavtoavg m-file of Brainstem tool box. The ‘.avg’ format of both the stimuli were band pass filtered from 30 to 3000 Hz using Neuroscan (Scan 2-version 4.4) to know the functional relationship between the acoustic structure of speech and the brain stem response to speech. The ‘stimulus.avg’, waveforms and spectrograms of the two CV tokens are depicted in Fig. 2. Table 2 summarizes the fundamental frequency (*F*_0_) and the first two formant frequencies (*F*_1_ and *F*_2_) of the vowel component of /da/ and /si/ stimuli. The onset to steady state *F*_0_, *F*_1_ and *F*_2_ within the transition duration (37 ms) of /da/ stimulus, and frequency components within the transition duration (42 ms) of /i/ portion of /si/ stimulus were measured using Praat (version 5.1.29) software.

**Table 2:** Fundamental frequency and the two formant frequencies (in Hz) at the transition duration of original and filtered version of /ɖa/ and /si/ stimuli.

Stimuli		*F*_0_ (Hz)		*F*_1_ (Hz)		*F*_2_ (Hz)	
		Onset	Steady state	Onset	Steady state	Onset	Steady state
Original version	/ɖa/	135.7	131.2	519.8	556.3	1822.4	1677.7
	/si/	145.7	137.5	345.4	308.8	2268.5	2451.5

Filtered version	/ɖa/	135.7	131.2	519.8	556.3		
	/si/	145.7	137.5	345.4	308.8		

*F*_0_, fundamental frequency; *F*_1_ and *F*_2_, first and second formant frequencies; *F*_2_, for filtered version is not applicable since the upper cut-off frequency of the filter was 2 kHz.

Further, Kannada passage developed by Sairam and Manjula^32^ was read out in normal vocal effort by a female speaker was recorded using Adobe Audition (version 3) software. This recorded passage was used to determine the acceptable noise level (ANL). A goodness test was performed in order to verify the quality of the recorded Kannada passage, in which ten listeners with normal hearing rated the passage for naturalness.

### Hearing aid

Digital Behind The Ear (BTE) hearing aid was used to record the output at the ear canal and at the auditory brainstem response from each participant. According to the technical specifications, frequency range of test hearing aid extended from 0.210 to 6.5 kHz. The peak full-on gain was 58 dB and high-frequency average full-on gain was 49 dB. The functioning of the hearing aid was ensured at the beginning of the data collection and repeated periodically during data collection.

## Procedure

Each participant was classified into good and poor hearing aid performer using the behavioural ANL test. The test hearing aid with custom ear mould was fitted to each participant and its gain was optimized. To optimize the hearing aid gain, six Ling's syllables were presented at a calibrated level of 65 dB SPL through the audiometer in a sound field. The gain and the frequency response of the hearing aid were manipulated for the audibility of each of six Ling's syllables, through fine tuning option. To know the extent to which spectral feature are preserved by the hearing aid, the output of the hearing aid to each stimulus was recorded at the ear canal using the probe tube microphone measure. Further, the FFR at the brainstem level was recorded to each stimulus, in both unaided and aided conditions.

### Acceptable noise level

Acceptable noise level (ANL) evaluates the reaction of the listener to background noise while listening to speech. For the measurement of ANL, the method given by Nabelek et al.^5^ was adopted. Each study participant was made to sit comfortably on a chair in front of the loudspeaker of the audiometer that was located at 1 m distance and 45° Azimuth. To compute the ANL, most comfort level (MCL) and background noise level (BNL) were measured.

The recorded Kannada passage was routed through the auxiliary input to the loudspeaker of the audiometer. The presentation level set at the level of SRT. Gradually, the level was adjusted in 5 dB-steps to establish the most comfortable level (MCL) and then in smaller steps size of +1 and −2 dB, until the MCL was established reliably. After the MCL was established, speech noise was introduced at 30 dB HL. The level of the speech noise was increased in 5 dB-steps initially, and then in 2 dB-steps, to a point at which the participant was willing to accept the noise without becoming tired or fatigued while listening to and following the words in the story. The maximum level at which he/she could accept or put up with the speech noise without becoming tired was considered as the background noise level (BNL). The level of the speech noise was adjusted until participant was able to ‘put-up-with’ the noise while following the story. The resultant level was the BNL. The ANL quantifies the acceptable level of background noise and is calculated as the difference between MCL (dB HL) and BNL (dB HL).^4^ Based on the ANLs, each participant was classified as good (ANL of ≤7 dB) or poor (ANL of ≥13 dB) hearing aid performers.^4^ The procedure of ANL was repeated twice and the average of the two values was considered as the ANL for each participant.

### Hearing aid gain optimization

Each participant was fitted with the digital BTE test hearing aid using a custom made soft shell mould. The hearing aid was programmed using NAL-NL1 prescriptive formula. The real ear measurement was carried out to match the gain of hearing aid with the target gain objectively. Further, the Ling's six speech sounds were presented at 65 dB SPL to optimize the hearing aid gain. Through fine tuning option, the gain and the frequency shaping of the hearing aid were optimized for the audibility of Ling's six sounds.

### Hearing aid processed speech at ear canal

The level of the each signal (stored in personal computer) was varied in the audiometer so that the intensity measured was 65 dB SPL in sound level meter. Larson Davis 824 sound level meter (SLM) was positioned at the test ear of the participant. The SLM was set at fast weighting function, and it was ensured that the stimuli /da/ and /si/ were presented at 65 dB SPL, based on peak amplitude level read on the SLM. After the calibration of stimulus was ensured, output spectrum at the ear canal was recorded using the probe tube microphone measurement, in both unaided and aided conditions. The probe tube microphone in the ear canal picks up the spectral energies at approximately half-octave bands from 0.25 kHz to 8 kHz for each speech stimulus. The levels as a function of frequency from 0.25 kHz to 8 kHz, in octaves, were noted down for each stimulus, in the unaided and aided conditions.

### Acquisition of the frequency following response

Each participant was seated comfortably in a reclining chair with arm. The electrode sites were cleaned up with skin preparing gel. Disc type silver coated electrodes were placed using conduction gel at the test sites. The FFR was recorded using vertical montage. The non-inverting electrode (+) was placed on the vertex (Cz), the ground electrode was on upper fore head (Fpz) and the inverting electrode (−) was placed on nose. It was ensured that the electrode impedance was less than 5 kΩ for each of the electrodes and that the inter-electrode impedance was less than 2 kΩ.

Prior to recording, calibration of stimuli was ensured using Larson Davis System 824 SLM. The SLM was positioned at reference point. It is the point where the test ear of the participant would be positioned at the time of testing. The SLM was set at fast weighting function for the measurement. It was ensured that both stimuli /da/ and /si/ were presented at 65 dB SPL, based on peak amplitude level read on the SLM.

The loudspeaker of the Auditory Evoked Potential equipment was placed at 45° Azimuth from the participant test ear, located at the calibrated position of 12 inch distance. The height of loudspeaker was adjusted to the level of participant test ear. The participant was instructed to ignore the stimulus and to watch a movie that was muted and played through a battery operated laptop computer. He/she was also asked to minimize the eye and head movement.

For recording the unaided and the aided FFR, the stimulus /da/ was presented through loud speaker at the presentation level of 65 dB SPL to the test ear. The PC-based evoked potential system, Neuroscan 4.4 (Stim 2-version 4.4), controlled the timing of stimulus presentation and delivered an external trigger to the evoked potential recording system, Neuroscan (Scan 2-version 4.4). To allow for a sufficient refractory period within the stimulus sweep, while minimizing the total recording time, an inter-stimulus interval (ISI) of 93 ms. from offset to onset of the next stimulus was included for recording FFR to /da/ stimulus. A similar procedure was repeated to record the unaided and aided FFR for /si/ stimulus. However, for recording unaided and aided FFR to /si/ stimulus, an ISI of 113 ms was used. The order of stimuli while testing on each participant was counter balanced. The FFR was recorded from 1500 sweeps each in condensation and rarefaction polarities, delivered in a homogenous train using the stimulus presentation software Neuroscan 4.4 (Stim 2-version 4.4).

The FFR recording was initiated once a stable electroencephalogram (EEG) was obtained. The ongoing EEG was converted from analogue-to-digital with the rate of 20,000 Hz. The continuous EEG was online band-pass filtered from 30 to 3000 Hz with 12 dB/octave roll-off. This was stored to disc for offline analysis.

### Data analyses

The output of the hearing aid in the ear canal for each stimulus in the unaided and aided conditions were analyzed for spectra. Further, the FFR recorded was analyzed for *F*_0_, *F*_0_ energy and *F*_1_ energy obtained for each stimulus. The continuous EEG data were epoched over a window of 160 ms for /da/ stimulus (which included a 30 ms pre-stimulus period and a 130 ms post-stimulus time). The response for /si/ stimulus was epoched over a window of 360 ms (which included a pre-stimulus period of 30 ms and a post-stimulus period of 330 ms). The epoched waveforms were corrected for baseline. The responses were averaged and filtered off-line from 0.030 kHz (high-pass filter, 12 dB/octave) to 3 kHz (low-pass filter, 12 dB/octave). All artefacts exceeding ±35 μV were rejected while averaging the response for each averaged response, in rarefaction and condensation polarity. A minimum of 1450 artefact-free epochs was ensured. The averaged waveforms of rarefaction and condensation polarities were added. Further, the added waveforms were created by averaging two trials recorded for each stimulus, in unaided and aided conditions.

**Figure 3:**
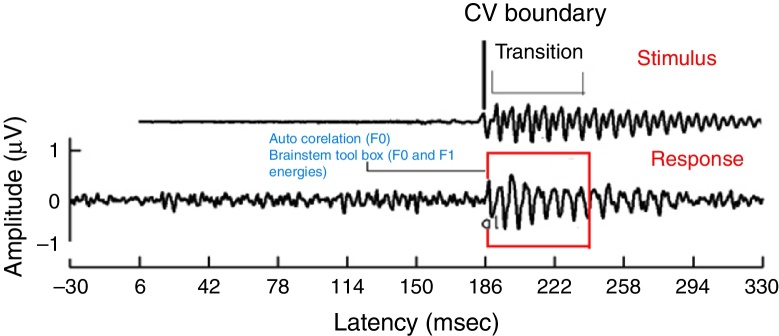
Transition response of FFR obtained from /si/ stimulus in aided condition.

**Figure 4:**
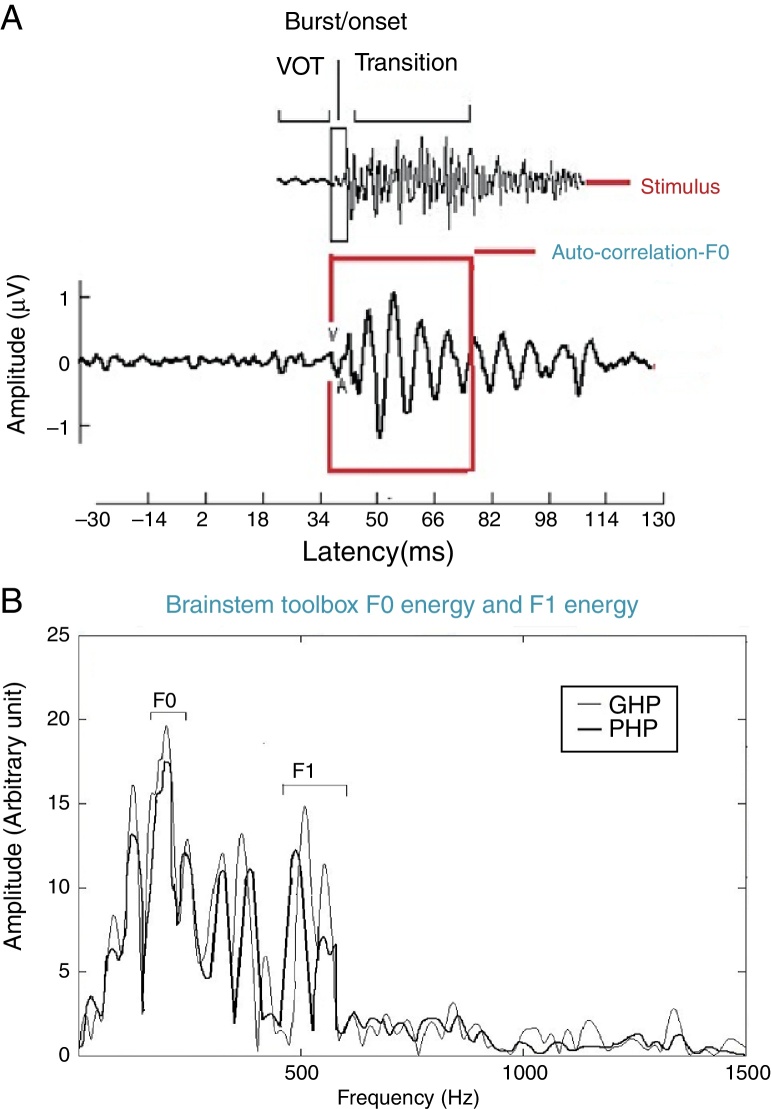
(A) Response corresponding to stimulus at transition portion of /da/ stimulus; (B) showing grand average spectrum of GHP and PHP sub-group. (B) Fundamental frequency and frequency of first formant in FFR for /da/ stimulus.

For all the participants, the unaided responses were absent for both the stimuli. From FFR recorded for /da/ stimulus in aided condition, the latency of ‘V’ peak was identified by visual inspection. The default MATLAB-code of autocorrelation was utilized, in which a range for latency was specified to obtain *F*_0_ in the FFR i.e., from noted ‘V’ peak latency till transition duration. Whereas, the latency of ‘a1’ corresponding to CV boundary^30^ in the FFR was identified for /si/ stimulus is shown in Fig. 3. The latency of ‘a1’ till transition duration was specified in autocorrelation MATLAB code to obtain *F*_0_ in the FFR. Further, *F*_0_ energy and *F*_1_ energy were determined, using ‘Brainstem Toolbox’ which utilizes the FFT technique (Fig. 4), from the transient response (‘V’ peak for /da/ stimulus; and ‘a1’ for /si/ stimulus) till specified transition duration (37 ms for /ɖa/ stimulus; and 47.1 ms for /si/ stimulus).^33^

## Results

The spectral data obtained at the ear canal using probe tube measurement and FFR at brainstem level were analyzed in good and poor hearing aid performers. Statistical Package for the Social Sciences (SPSS for window, version 17) software was used to perform the statistical analyses. The results obtained are discussed with respect to each objective.

### Hearing aid output at the ear canal

**Figure 5:**
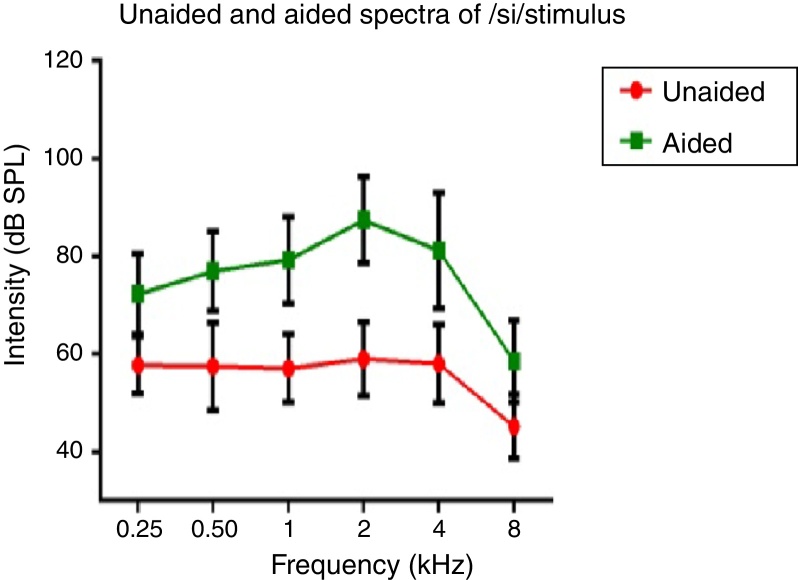
Mean and standard deviation of intensity of /si/ stimulus as a function of frequency in unaided and aided conditions.

**Figure 6:**
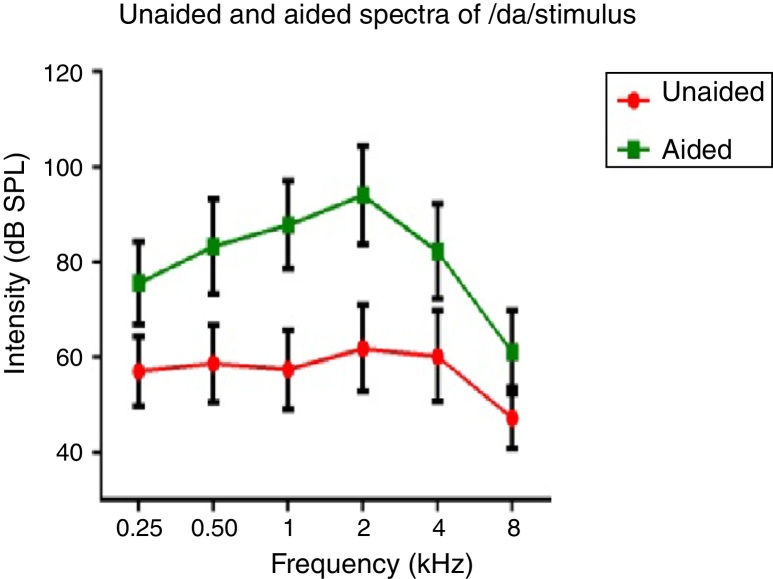
Mean and standard deviation of intensity of /ɖa/ stimulus as a function of frequency in unaided and aided conditions.

Spectral energy at frequencies from 0.25 to 8 kHz (in octaves) in the unaided and aided conditions, for both the stimuli was analyzed. It was performed to determine representation of energy across frequencies at the ear canal, in good and poor hearing aid performers. The data of spectral energy met the assumption of normal distribution on Kolmogorov–Smirnov normality test (*p* > 0.05) and homogeneity on Levene's test (*F* < 2). The spectral energy (0.5–8 kHz in octaves) for both stimuli obtained from both groups, in unaided and aided conditions, was subjected to MANOVA. The result revealed that there was no significant difference between groups in the spectral energy at each octave frequency, in both the unaided and the aided conditions, for /ɖa/ and /si/ stimuli. Thus, the data of spectral energy was combined between groups. Descriptive analysis was carried out separately in the unaided and aided conditions. For /ɖa/ stimulus (Fig. 5), at extreme low frequency (0.25 kHz) and at extreme high frequencies (4 kHz and 8 kHz) the energy in both unaided and aided conditions is relatively minimal than at other frequencies (0.5 kHz, 1 kHz and 2 kHz). For /si/ stimulus (Fig. 6), at extreme low frequencies (0.25 kHz) and at extreme high frequency (8 kHz) the energy in both unaided and aided conditions is relatively minimal compared to other frequencies (1 kHz, 2 kHz and 4 kHz).

### Comparison of FFR in terms of *F*_0_, *F*_0_ energy and *F*_1_ energy in good hearing aid performers and poor hearing aid performers

**Figure 7:**
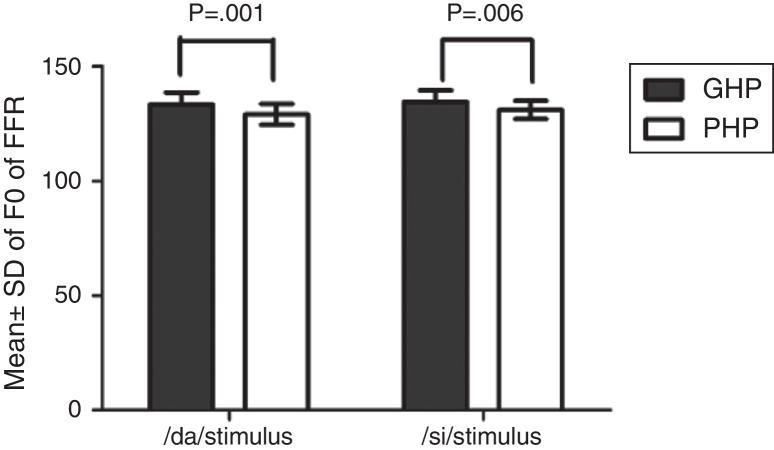
Mean, standard deviation and *p*-value of independent samples *t*-test on *F*_0_ of FFR for each stimulus, in GHP and PHP groups.

The *F*_0_, *F*_0_ energy and *F*_1_ energy of FFR between the groups for each stimulus met the assumption of normal distribution on Kolmogorov–Smirnov normality test (*p* > 0.05) and homogeneity on Levene's test (*F* < 2) was also performed. Hence, an independent samples *t*-test was conducted on each data of FFR between GHP (*n* = 34) and PHP (*n* = 24) groups. From the mean value of *F*_0_ of FFR (Fig. 7), it can be inferred that the *F*_0_ was represented better in GHP than in PHP, for each stimulus. Further, the *F*_0_ of FFR was compared between GHP and PHP using independent samples test. The result showed that there was a significant better *F*_0_ encoding in GHP than PHP for /da/ stimulus (*t* = 3.41, *p* = 0.001) and /si/ stimulus (*t* = 2.84, *p* = 0.006).

**Figure 8:**
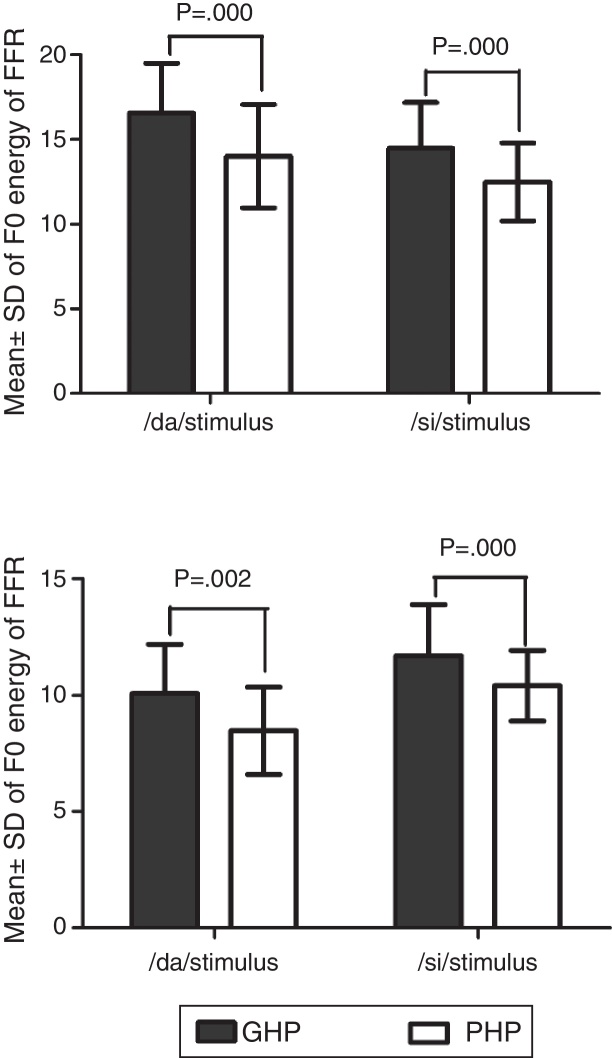
Mean and standard deviation of *F*_0_ energy and *F*_1_ energy for each stimulus in GHP and PHP groups.

In addition, the *F*_0_ energy and the *F*_1_ energy of FFR was compared between GHP and PHP groups to each stimulus. From Fig. 8, it was noted that the mean and standard deviation of *F*_0_ energy and *F*_1_ energy of FFR to each stimulus were higher in GHP than in PHP. Further, to know if there was any significant difference between GHP and PHP in the mean the *F*_0_ energy and the *F*_1_ energy of FFR for each stimulus, independent samples t-test was performed. The result revealed a significant higher *F*_0_ energy in GHP than in PHP for /da/ stimulus (*t* = 6.80, *p* = 0.000) and /si/ stimulus (*t* = 6.20, *p* = 0.000). Further, a significant higher *F*_1_ energy was observed in GHP than PHP for /da/ (*t* = 3.11, *p* = 0.002) and /si/ stimulus (*t* = 5.20, *p* = 0.000).

## Discussion

The aim of the study was to investigate the representation of amplified speech at the ear canal and at the auditory brainstem from the good and the poor hearing aid performers.

### Effect of hearing aid processing on spectral parameters of speech stimuli

In the unaided condition for /da/ and /si/ stimuli, energy measured at 2 kHz was relatively larger than at other frequencies (in octave). Further, there was a decline in energy after 4 kHz i.e., an approximately 10 dB per octave for /da/ stimulus and 12 dB per octave for /si/ stimulus (Figure 5, Figure 6). This pattern of energy representation as a function of frequencies, for both the stimuli, could be because of frequency response of microphone used in recording the target test stimuli. In the aided condition for /da/ stimulus and /si/ stimulus, the energy measured was relatively less at the two extreme cut off frequencies, which is at low frequency below 0.25 kHz and high frequencies above 4 kHz. Thus, at extreme frequencies, the mean energy in both unaided and aided conditions was less. At low frequency, reduced energy could be because of less gain in that frequency region provided by the prescriptive formula.^34^ Additionally, low energy noted in the low frequency region of /da/ and /si/ stimulus could also be because of frequency response of the hearing aid. The low frequency cut-off of the frequency response of the test hearing aid was 0.21 kHz. At high frequencies i.e., above 4 kHz, the energy reduced approximately at the rate of 10 dB per octave for /da/ and 14 dB per octave for /si/ stimulus. This could be the frequency response of /da/ and /si/ stimulus had energy till 4 kHz as noted from unaided condition. Yet another reason could be though the frequency response of hearing aid had 0.216 to 6.5 kHz, energy after 4 kHz gradually reduced per octave. Thus, remarkable energy was noted in the frequency range from 0.5 kHz to 4 kHz. It can be inferred that there is a relatively high amplification in the mid-frequency region of the hearing aid than other two extreme cut-off frequencies (low and high). Informally, participants were instructed to repeat the syllables which were randomly presented for three times. In unaided condition, the participants were unable to identify the CV tokens as the presentation level was 65 dB SPL, which failed to reach audibility range. However, in aided condition, all the participants consistently identified syllables. Further, on spectral analysis, it was noted that the amplitude of aided burst spectrum of /da/ was similar to the unprocessed burst spectrum amplitude of /da/. It was also observed that amplitude spectrum of fricative /si/ was similar to the unprocessed fricative spectrum amplitude of /si/. This infers that hearing aid preserves inherent speech cues at the ear canal.

### Comparison of *F*_0_ of FFR, *F*_0_ energy and *F*_1_ energy in good and poor hearing aid performers

The FFR in both unaided and aided conditions were obtained from all the participants. In the unaided condition, the brainstem responses were absent, as the stimuli (/da/ and /si/) were presented at 65 dB SPL, which failed to reach audibility. In the aided condition, the *F*_0_ representation in the FFR to each stimulus (/da/ and /si/) remained robust and similar to that of the unprocessed filtered raw stimulus. This indicated that preserved spectral content from hearing aid is relayed to the auditory brainstem level. For /da/ stimulus, the mean *F*_0_ of FFR was higher in GHP (133.46 Hz) than in PHP (128.84), such that the difference was found to be significantly different. This was true for *F*_0_ of FFR for /si/ stimulus between GHP (134.42 Hz) and PHP (130.84 Hz). Further, the *F*_0_ of the aided stimulus of /da/ was 134.95 Hz and that for /si/ was 144.74 Hz. The difference in *F*_0_ (in Hz), between encoding of *F*_0_ at brainstem level and *F*_0_ of aided test stimulus was 1 Hz in GHP and 6 Hz in PHP for /da/ stimulus. Similarly, the difference noted was 10 Hz in GHP and 14 Hz in PHP for /si/ stimulus.

The mean difference between the GHP and PHP in the encoding of *F*_0_ was 5 Hz for /da/ and 4 Hz for /si/ stimulus. Though this difference was significant in the encoding of *F*_0_ between GHP and PHP for both stimuli, this may not bring a change in speaker identity. This is because, according to Iles^35^ a change of up to ±25 Hz in the *F*_0_ will not bring about a change in speaker identity. The finding of the study is in accordance with the research report by Horii^36^ who reported that a difference of greater than 25 Hz in the *F*_0_ between the same two stimuli does not cause difference in speaker identity. Additionally, the intra-subject variability of *F*_0_ in a normal vocal effort ranged between ±9.6 Hz.^37^ Thus, it can be inferred that the mean *F*_0_ of FFR to /da/ and /si/ stimuli was neurally well represented in GHP than PHP, and that both the groups were able to recognize the identity of the speaker.

Further, it was noted that the *F*_0_ energy and the *F*_1_ energy of FFR to each stimulus were significantly higher in GHP than PHP. The higher energies of *F*_0_ and *F*_1_ in GHP might be due to stronger efferent fibres that inhibit other harmonics that do not correspond to fundamental frequency and formant frequencies. This is in accordance with the research reports by Ashmore^38^ and Knight.^39^ To be more specific, central afferent mechanism is stronger in the group of GHP such that neurons at inferior colliculus fire precisely to the harmonics corresponding to *F*_0_ and *F*_1_. In addition, the efferent mechanism might be stronger such that the efferent fibres inhibit the other harmonics which do not correspond to the fundamental frequency and formant frequencies, thereby fine tuning the auditory input. The excitatory and inhibitory mechanisms of neurons of the underlying neural generator of the inferior colliculus in GHP fire more or less precisely to the corresponding *F*_0_ and *F*_1_ components of the stimulus. The inference of the present study supports the findings reported by Krishnan.^40^ He demonstrated that efferent auditory pathway suppresses energies adjacent to the harmonics corresponding to the *F*_0_ and the *F*_1_ of FFR. Along with an active afferent pathway, the afferent auditory nerve generates the electrical activity more precisely corresponding to the *F*_0_ and the *F*_1_ of the stimulus. This involves the release of neurotransmitter, thereby reducing the trans-membrane threshold and increase in neural firing. In poor hearing aid performers, though similar physiological activity was present, probably a lack of precision in neural activity due to less sensitive afferent and weak efferent auditory pathway, might have failed to provide higher energy at harmonics corresponding to *F*_0_ and *F*_1_ of each stimulus. Thus, it is can be inferred from the present study that subtle physiological variations might be present at the inferior colliculus of the auditory pathway in the poor hearing aid performers with reference to that in good hearing aid performers.

## Conclusion

Though the hearing aid preserved inherent cues in speech syllables, an effect of annoyance towards noise alters the neural encoding at auditory brainstem level. It infers that acoustic cues transferred by hearing aid are successfully relied at auditory brainstem level but subtle physiological alterations were present at auditory brainstem in those individuals who are annoyed from those who are not by noise.

## Implication

The study presents an evidence to use objective approaches to validate the hearing aid output at ear canal and at auditory brainstem level. Utilization of the real ear measurement for analyzing the hearing aid output in the ear canal will help in knowing the representation of inherent speech cues. Studying the encoding of amplified speech in individuals with hearing impairment with their annoyance level demonstrates a critical role of stimulus contingent response in the assessment of hearing aid algorithms. It solves some of the practical problems faced by the audiologists regarding setting of amplification parameters in providing the maximum usable information. Findings of the present study help the audiologist in counselling a hearing aid user regarding extent of benefit derived with best hearing aid prescribed.

## Conflicts of interest

The authors declare no conflicts of interest.
